# Experimental Study of Airworthiness Compliance Verification of High-Temperature Environment in Aircraft Cockpit

**DOI:** 10.3390/s25030764

**Published:** 2025-01-27

**Authors:** Haiming Shen, Jiawei Ren, Hao Shen, Weijian Chen, Zhongchao Hua

**Affiliations:** 1 Key Laboratory of Civil Aircraft Airworthiness Technology, Civil Aviation University of China, Tianjin 300300, China; 2 College of Safety Science and Engineering, Civil Aviation University of China, Tianjin 300300, China; 19851623325@163.com (J.R.); 15061807867@163.com (Z.H.); 3 Shanghai Aircraft Airworthiness Certification Center, CAAC, Shanghai 200335, China; shenhao_hd@caac.gov.cn; 4 Key Laboratory of Aircraft Environment Control and Life Support, Ministry of Industry and Information Technology, Nanjing University of Aeronautics and Astronautics, Nanjing 210016, China; weijian_chen@nuaa.edu.cn

**Keywords:** high temperature, core temperature, physiological parameters, airworthiness compliance

## Abstract

The aim of this study was to assess the applicability of the Mechanical Systems Coordination Working Group’s (MSCWG) findings, based on FAR 25.831(g), to Chinese pilots through a human physiological experiment conducted in a high-temperature environment to investigate the effects of core temperature. Methods: A controlled experiment was carried out in a high-temperature environment simulation room involving a cohort of healthy males aged 18–50 years. Wireless physiological monitoring equipment and a neurobehavioral assessment system were utilized to track changes in physiological parameters and neurobehavioral responses at varying core temperatures and time intervals. Results: There was a significant increase in human core body temperature, skin temperature, and heart rate as the ambient temperature rose, all remaining within acceptable physiological limits. Although arterial and venous oxygen saturation decreased with increasing ambient temperature, the difference was not statistically significant. The neurobehavioral abilities of the subjects did not exhibit notable changes across different core temperature–time conditions. Conclusions: The core temperature limits set forth by the MSCWG have been shown to have a safe impact on the physiological and behavioral aspects of Chinese pilots, which can be used as an equivalent safety regulation for airworthiness compliance validation under CCAR 25.831(g). Limitation: The present study was constrained to a male sample, it did not thoroughly explore female responses, and it had a small sample size (10 per group). The latter two factors may have affected the statistical validity and generalizability of the results.

## 1. Introduction

When the cockpit environmental control system fails, the temperature and humidity conditions in the cabin may rise and exceed the tolerance limit of the pilot, and the pilot may bear a large thermal load and enter different degrees of thermal stress, which may pose a threat to flight safety. Thermal stress is one of the important factors leading to flight accidents [1]. The high temperature conditions in the cockpit may affect the pilot’s cognitive judgment ability, thus affecting his ability to control the aircraft. Some studies have pointed out that the higher the thermal intensity of the cabin environment, the greater the psychological and physiological load of the operators, and the higher the risk of fatigue and injury [2,3].

**Figure 1:**
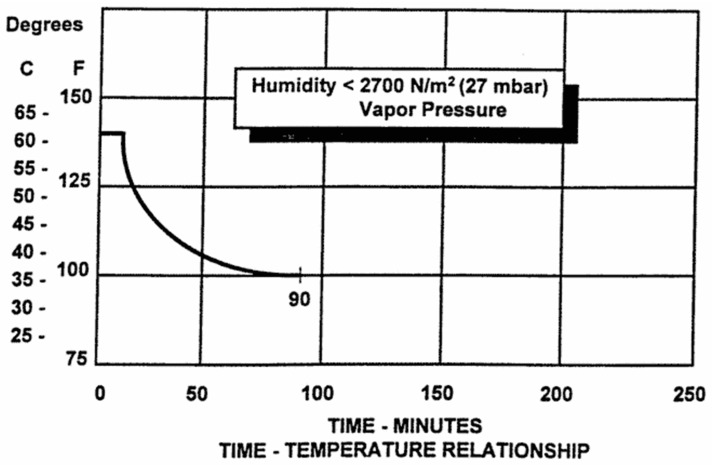
Time–temperature relationship curve.

China has formulated the corresponding airworthiness clause CCAR-25.831(g) according to the FAR25 of the United States. As shown in Figure 1, the clause stipulates: ‘After any failure with small probability occurs, the duration at a given temperature shall not exceed the specified value, and it is also required to keep the humidity in the aircraft below 27mbar’ [4]. Using this criterion, Zhou’s study found that after 55 min in a hot and humid environment—where fighter pilots should not operate for more than 55 min—the pilot’s core temperature reached 38 °C [5].

The purpose of this regulation is to limit the exposure time of the cockpit and cabin to a high-temperature environment, so as to ensure that even if the ventilation system fails, the aircraft can still fly and land safely, and at the same time avoid causing physiological harm to the crew and passengers. However, the manufacturers found that it was difficult or impossible to meet the requirements of 25.831(g), and they could not meet the airworthiness compliance when all gas supply was lost due to the failure of the environmental control system. Therefore, ARAC assigned this task to the Mechanical System Coordination Working Group (MSHWG), and MSHWG released their final report on 31 July 2003, in which a new performance-based standard was formulated to maintain a tolerable environment by limiting crew metabolism and environmental heat load during possible thermal stress exposure.

The final report of MSHWG gives the following equivalent safety rules [6]: ‘The aircraft design must adapt to any failure situation of the environmental control system that is not indicated to be extremely impossible. The following failure situations apply: (a) the environmental conditions of the cockpit and cabin must not adversely affect the crew’s work, thus leading to dangerous situations; (b) No occupant will suffer permanent physical injury. By verifying the above rules, it can be indirectly proved that the aircraft meets the airworthiness clause CCAR25.831(g). The purpose of this rule is to ensure that the cockpit environment will not affect the crew’s work efficiency, to avoid causing cognitive dysfunction or physical fatigue, and to ensure that they can complete the flight mission safely. It will not cause harm to ordinary sit-in passengers. The acceptable way to comply with this rule is the following: For the cockpit environment when it fails, it is proved through analysis that the crew’s work efficiency will not be affected and the passengers’ health will not be harmed.

For the above proposed regulations, MSHWG proposed the criteria for judging the acceptable high-temperature environment in the cabin based on the core temperature of the human body, requiring the applicant to ensure that when the air-conditioning system is not installed or fails, the core temperature of the human body does not exceed 38 °C (100.4 degrees Fahrenheit) in the steady state; that the temperature does not exceed 38.5 °C (101.3 °C, Fahrenheit) under transient conditions of not more than 20 min; and that the human core temperature of 38.5 °C cannot exceed or last for any length of time [7]. Studies have shown that when the ambient temperature is too high, the heat storage rate of the human body is greater than zero, and if the residual heat cannot be dissipated in time, then the human body will feel uncomfortable, and when the body temperature reaches 38.2 °C, mild heatstroke may occur [8]. According to the research results of MSHWG on 25.831(g), the influence of the increase in human core temperature on the crew and passengers is shown in Table 1.

However, this standard is based on the medical research results of western ethnic groups [9,10,11]. Although some studies related to human functions in a high-temperature environment have been carried out in China [12,13], there is a paucity of research findings regarding the applicability of these criteria to the Chinese pilot situation. Therefore, this study takes the general population of different ages as the experimental object, analyzes the core temperature values of the human body in different cabin temperature environments, evaluates the changes in their physiological response and cognitive function, and verifies the accuracy of Table 1 through statistical data. The purpose of this study is to verify the acceptable high-temperature environment standard in the cabin based on the core temperature of the human body, which can be used as a feasible cockpit environment equivalent safety analysis method and provide strong support for the equivalent safety verification of the airworthiness clause CCAR25.831(g).

## 2. Subjects and Methods

### 2.1. Subjects

Members of the general population aged 18–50 years old were selected as experimental subjects, with 10 people in each group. The general population was divided into three groups according to age: group 20 to 30 years old (age 24.00 ± 1.11 years old, BMI index 21.75 ± 2.13), group 30 to 39 years old (age 32.90 ± 2.06 years old, BMI index 24.02 ± 2.12), group 40 years old and above (age 44.63 ± 3.74 years old, BMI index 22.18 ± 2.48). Due to the specific characteristics of pilots, who are predominantly male, the study included only Chinese males who were in good health, had no medical history, had never been to a high-altitude area, had a physical examination within 3 months before the experiment, and had good sleep and diet before the experiment. The present experiment focuses on the relative changes of subjects in the high-temperature environment rather than on the absolute values. Changes in physiological parameters and cognitive abilities before and after the experiment can better reflect the effects of the high-temperature environment on the subjects, rather than relying solely on the initial state. Thus, even if the subjects differ at baseline, the final outcome can still be assessed by relative change. The study was formally reviewed and endorsed by the Ethics Committee of Soochow University on 1 March 2019, affirming that the rights and well-being of the subjects were thoroughly safeguarded throughout the study and that there was no inherent risk to the subjects.

### 2.2. Experimental Methods

In this study, we measured human heat load in terms of core temperature according to MSHWG regulations and designed three groups with different levels of heat load to systematically explore physiological responses under different conditions. The specific experimental groups were as follows: the first group prior to entering the hot environment (referred to as pre-experiment), the second group after the core body temperature reached 38 °C, and the third group after a duration of 20 min between 38 °C and 38.5 °C.

The high-temperature environment was simulated by a walk-in artificial climate chamber (ESPEC, Osaka, Japan). The core temperature acquisition system (HQinc, Los Angeles, CA, USA) was used in the experiment to monitor the core temperature (Tcore). Metabolism was monitored with a cardiopulmonary function meter (Cortex, Düsseldorf, Germany); a portable heart rate band (Polar, Oulu, Finland) was used to monitor the heart rate (HR), and a wireless oxygen monitoring system (Artinis, Nijmegen, The Netherlands) was used to collect the saturation of arterial blood oxygen (SAO2) and saturation of venous blood oxygen (SVO2); the MSR145WD wireless data logger (MSR, Baar, Switzerland) was used to collect skin temperature (TSK). Neurobehavioral ability indexes (mental arithmetic, visual retention, attention transfer, and selection response) were evaluated by the third edition of the Chinese Neurobehavioral Assessment System (NES-C3, Shanghai, China), and the Neurobehavioral Ability Index (NAI) was calculated. The Brog scale and the Rating of Perceived Exertion (RPE) were used to evaluate the degree of fatigue.

The experimental conditions were set as follows: temperature 40 ± 0.5 °C, relative humidity 40 ± 5% RH, wind speed 0.2 ± 0.1 m/s. The present experiment focuses on the effects of heat load on the body in a hot environment. Provided that the variable of high temperature is effectively controlled, even though barometric pressure and noise may affect the occupants during actual flight, these differences will not affect the main results and conclusions of the experiment. The subjects walked on a treadmill at an incline speed of 5 km/h. The experiment employed subjects walking on a treadmill for two primary purposes. First, this simplified and simulated the workload of the pilot in the event of a ventilation system failure during flight, thereby rendering the experiment more realistic. Secondly, this approach enabled the expeditious attainment of the stipulated conditions for the subjects, thereby facilitating the execution of the experiment. The experimental process is as follows:

(1) First, the thermal neutral state was reached in the room temperature environment in advance, and the neurobehavioral ability of the subjects was assessed by an NES-C3 system.
(2) The subjects changed the test clothes (ordinary long sleeves and trousers), wore the test equipment of physiological indicators, and then entered the climate chamber with a preset environment to prepare for the test.
(3) After the test began, the subjects walked on a treadmill at an incline speed of 5 km/h, recorded their subjective feelings, and measured their metabolic rate 15 min after the test began.
(4) The core body temperature of the subjects was monitored. When the core temperature of the subjects reached 38 °C, NES-C3 was used to evaluate the neurobehavioral ability of the subjects.
(5) The test was repeated after 20 min, and the core temperature was maintained at 38~38.5 °C during the test.

### 2.3. Data Processing

SPSS 26.0 statistical software was used to conduct a one-way ANOVA on the data of the four groups under different levels of heat load. All the data were expressed as mean ± standard deviation (x ± s). The significance level of difference was set at *p* < 0.05 *, and *p* < 0.01 ** indicates a very significant difference.

## 3. Results

### 3.1. Changes of Physiological Indexes Under Different Levels of Heat Load

#### 3.1.1. Changes in Core Body Temperature (Tcore) Under Different Levels of Heat Load

As shown in Table 2, with the increase in ambient temperature, the human core body temperature also increases. The experimental results show that the core body temperature of the subjects reached 38 °C in about 40 min and remained at about 38.5 °C at the end of the experiment. These values are all within the range of human physiological safety tolerance. In each age group, there were statistically significant differences between the different heat load environments (*p* < 0.01).

#### 3.1.2. Changes in Skin Temperature (TSK) Under Different Levels of Heat Load

**Figure 2:**
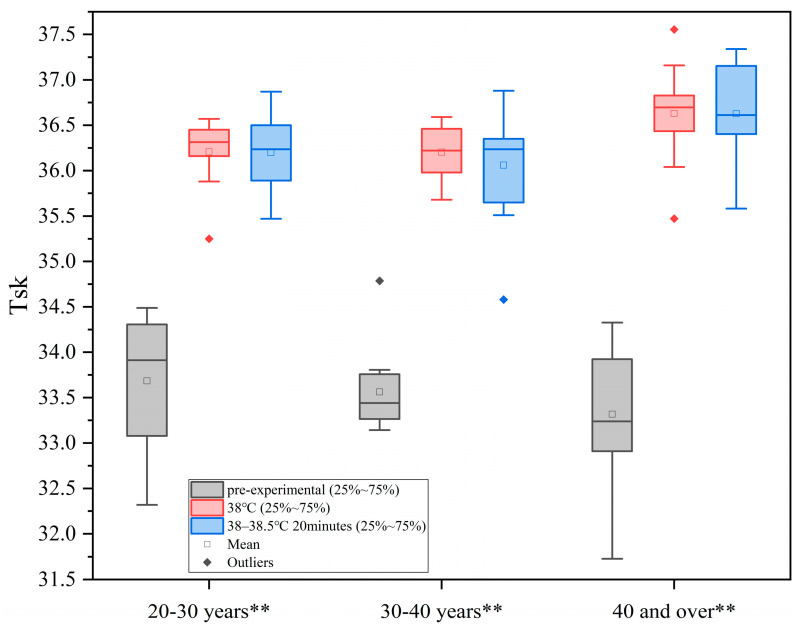
Changes in TSK under different levels of heat load.

As shown in Figure 2, with the increase in ambient temperature, human skin temperature increases and remains at about 36.5 °C, but these values are within the range of human physiological safety tolerance. In each age group, there were statistically significant differences between the different heat load environments (*p* < 0.01, Each group in the figure is represented by **).

Based on the results of the ANOVA, a linear regression analysis of TSK was conducted to investigate the relationship between heat load (x) and TSK ($y_{1}$). The analysis yielded the following relational equation: $y_{1}=-53.395+2.345x$. The regression coefficient was statistically significant (*p* < 0.05), and the model demonstrated a good fit (R^2^ > 0.6), as shown in Table 3. These findings indicate a significant linear relationship between heat load (x) and TSK ($y_{1}$).

#### 3.1.3. Changes in Heart Rate (HR) Under Different Levels of Heat Load

**Figure 3:**
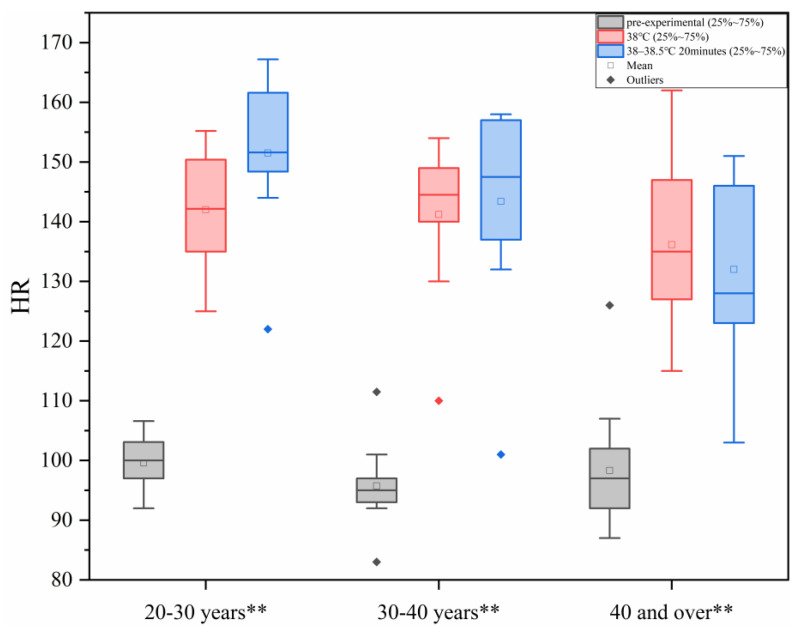
Changes in HR under different levels of heat load.

As shown in Figure 3, with the increase in ambient temperature, human heart rate increases, and these values are within the range of human physiological safety tolerance. In each age group, there were statistically significant differences between the different heat load environments (*p* < 0.01, Each group in the figure is represented by **).

Based on the results of the ANOVA, a linear regression analysis of HR was conducted to investigate the relationship between heat load (x) and HR ($y_{2}$). The analysis yielded the following relational equation: $y_{2}=-1241.765+36.123x$. The regression coefficient was statistically significant (*p* < 0.05), and the model demonstrated a good fit (R^2^ > 0.6), as shown in Table 4. These findings indicate a significant linear relationship between heat load (x) and HR ($y_{2}$).

#### 3.1.4. Changes in Arterial Oxygen Saturation (SAO_2_) Under Different Levels of Heat Load

**Figure 4:**
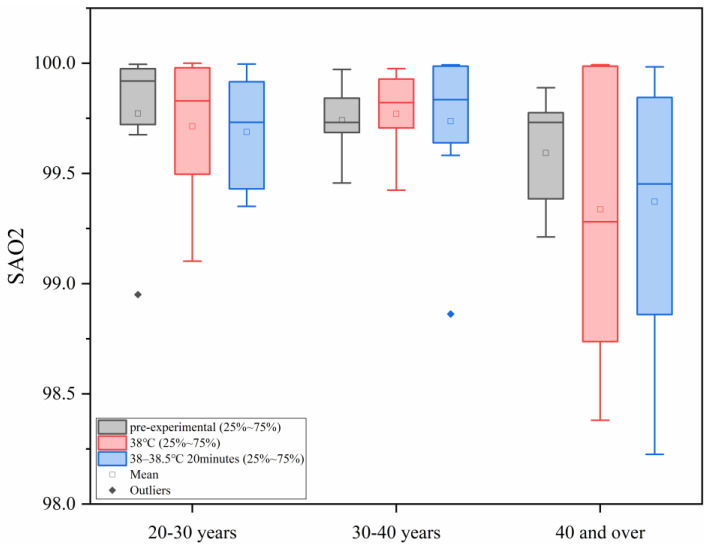
Changes in SAO2 under different levels of heat load.

As shown in Figure 4, with the increase in ambient temperature, human arterial oxygen saturation showed a downward trend. However, in each age group, the differences between the different heat load environments were not statistically significant. The final arterial oxygen saturation values were all within the safe tolerance range of human physiology.

#### 3.1.5. Changes in Venous Blood Oxygen Saturation (SVO_2_) Under Different Levels of Heat Load

**Figure 5:**
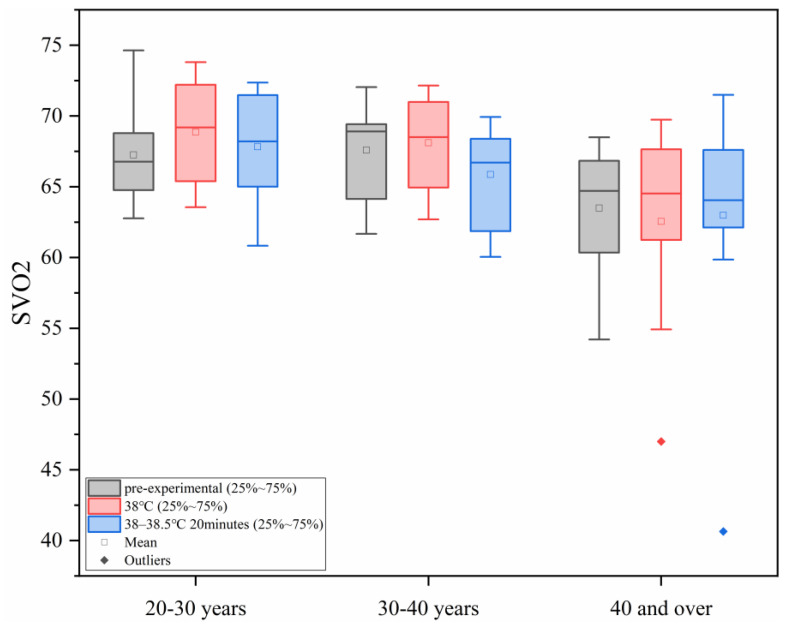
Changes in SVO2 under different levels of heat load.

As shown in Figure 5, with the increase in ambient temperature, the oxygen saturation of human venous blood tends to decrease. However, in each age group, the differences between the different heat load environments were not statistically significant. The final venous oxygen saturation values were all within the safe tolerance range of human physiology.

#### 3.1.6. Change in Fatigue Degree Under Different Levels of Heat Load

**Figure 6:**
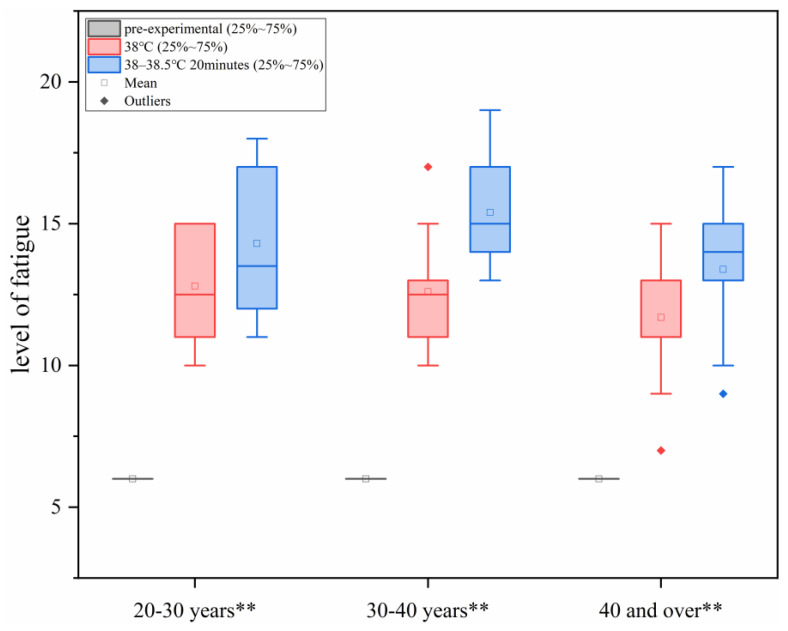
Changes in fatigue degree under different levels of thermal load environment.

As shown in Figure 6, with the increase in ambient temperature, the fatigue degree of the human body increased, and in each age group, there were statistically significant differences between the different heat load environments (*p* < 0.01, Each group in the figure is represented by **).

Based on the results of the ANOVA, a linear regression analysis of fatigue degree was conducted to investigate the relationship between heat load (x) and fatigue degree ($y_{3}$). The analysis yielded the following relational equation: $y_{3}=-233.002+6.439x$. The regression coefficient was statistically significant (*p* < 0.05), and the model demonstrated a good fit (R^2^ > 0.6), as shown in Table 5. These findings indicate a significant linear relationship between heat load (x) and fatigue degree ($y_{3}$).

### 3.2. Changes in Neurobehavioral Ability Indexes Under Different Levels of Heat Load

#### 3.2.1. Changes in Mental Arithmetic NAI Under Different Levels of Heat Load

**Figure 7:**
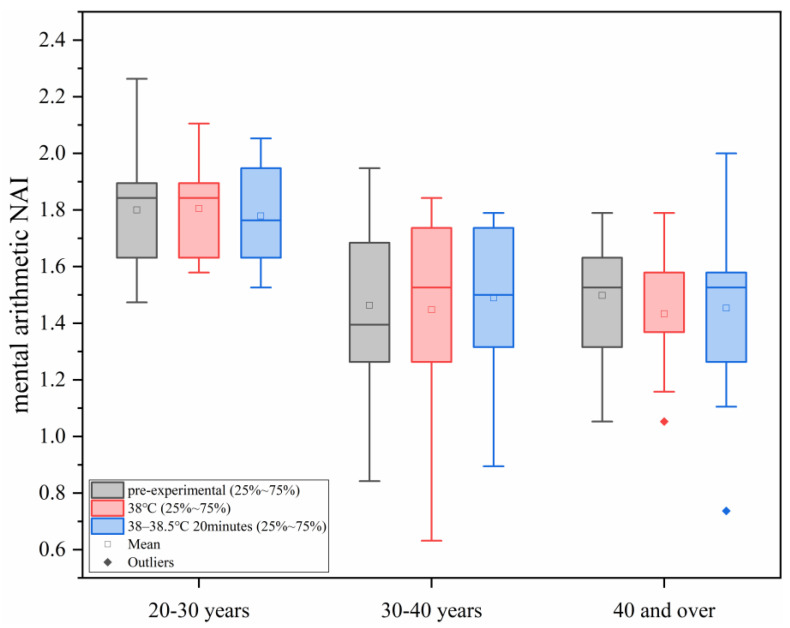
Changes in mental arithmetic NAI under different levels of heat load.

As shown in Figure 7, with the increase in ambient temperature, the study found that under different conditions of human core temperature–time, the NAI value of the mental arithmetic ability of all the age groups did not show significant changes, and in each age group, the differences between the different heat load environments were not statistically significant (*p* > 0.05).

#### 3.2.2. Changes in Visual Retention NAI Under Different Levels of Heat Load

**Figure 8:**
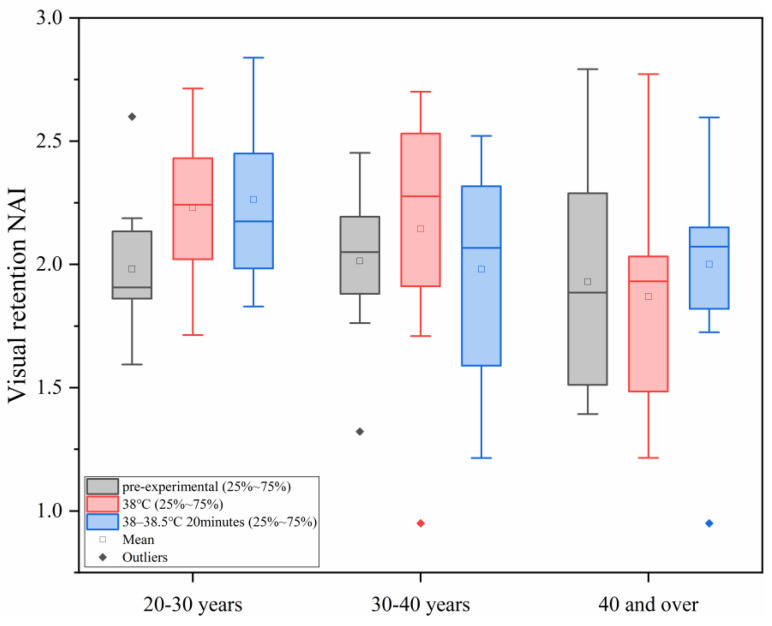
Changes in visual retention NAI under different levels of heat load.

As shown in Figure 8, with the increase in ambient temperature, the study found that under different conditions of human core temperature–time, the NAI value of visual retention ability of all the age groups did not show significant changes, and in each age group, the differences between the different heat load environments were not statistically significant (*p* > 0.05).

#### 3.2.3. Changes in Attention Transfer NAI Under Different Levels of Heat Load

**Figure 9:**
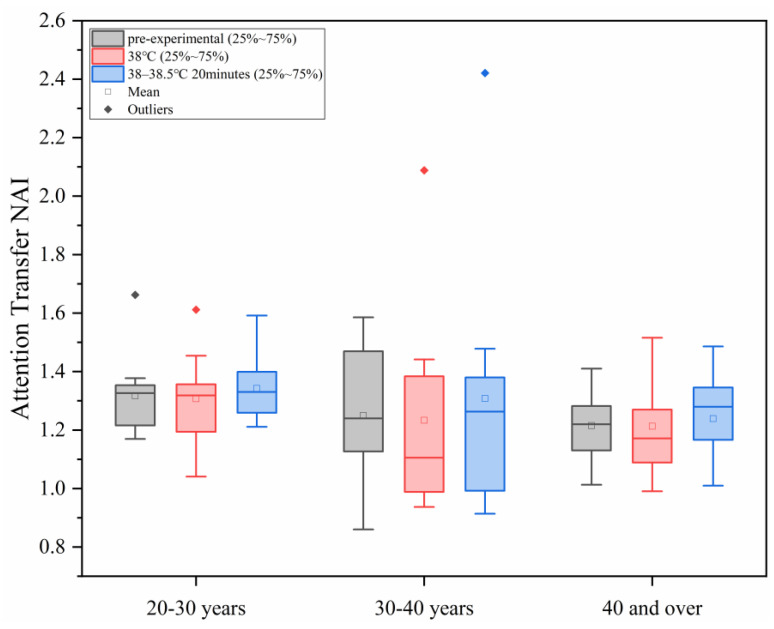
Changes in attention transfer NAI under different levels of heat load.

As shown in Figure 9, with the increase in ambient temperature, the study found that under different conditions of human core temperature–time, the NAI value of attention transfer ability of all the age groups did not show significant changes, and in each age group, the differences between the different heat load environments were not statistically significant (*p* > 0.05).

#### 3.2.4. Changes in NAI During Selection Response Under Different Levels of Heat Load

**Figure 10:**
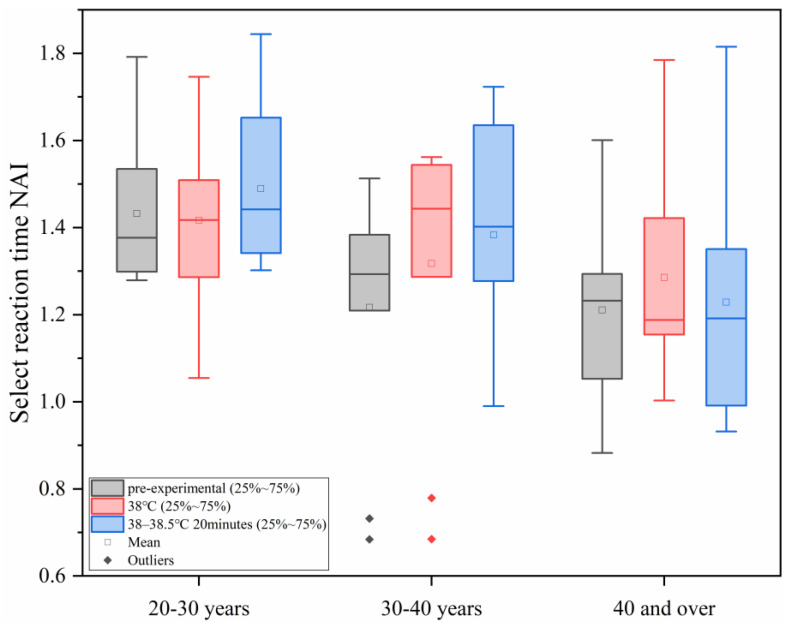
Changes in NAI in selective reaction under different levels of heat load.

As shown in Figure 10, with the increase in ambient temperature, the study found that under different conditions of human core temperature–time, the NAI value of the ability to select a response in all age groups did not show significant changes, and in each age group, the differences between the different heat load environments were not statistically significant (*p* > 0.05).

## 4. Discussion

### 4.1. Physiological Response Variations Under Different Heat Load Environments

The high-temperature environment chosen for this study was set at 40 °C, which falls within the range of 38–40 °C typically observed in the cockpit following a failure of the airplane’s air conditioning system [14]. This selection reflects a plausible real-world scenario. The safe range of human core temperature is in the range of 36–42 °C, and it is generally believed that if it exceeds 42 °C, there will be symptoms of heatstroke and even risk of death. The skin temperature of human body usually varies in the range of 32–37 °C, and when the skin temperature exceeds this range, it may cause discomfort or health problems. Studies have shown that the physiological tolerance limit of the human heart rate is 172 to 182 beats/min at the effective temperature of 35.4 to 38.5 °C [15]. After reaching the set temperature conditions, the conclusion of this experiment is consistent with the influence of the existing indoor thermal discomfort environment on human physiological response [16]. With the increase in temperature, the core temperature, skin temperature, and heart rate of the human body have significantly increased, while blood pressure and oxygen saturation have decreased, but they are all within the safe range. At the same time, a high-temperature operation can reduce the function and adaptability of the cerebral cortex, cause fatigue, and lead to operational errors [17]. The experimental results show that the fatigue degree of people in all age groups increases significantly with the increase in temperature, and the indexes of neurobehavioral ability change to some extent in a high-temperature environment, but there is no significant difference among various physiological indexes.

The present study revealed that skin temperature and heart rate exhibited a substantial response to heat load in pilots. Skin temperature is influenced by a combination of blood flow and blood temperature. An increase in ambient temperature results in the relaxation of blood vessels and the subsequent rise in blood flow, leading to an elevated skin temperature. Zhou’s experiments on flight simulation demonstrated a substantial increase in the average skin temperature of the human body with rising ambient temperature, accompanied by an increase in fatigue, which is consistent with the findings of the present study [5]. The rise in mean heart rate in a hot environment is predominantly attributed to cardiac sympathetic excitation. The observed rise in heart rate with core body temperature is designated as thermal cardiac reactivity (TCR). Consequently, skin temperature and heart rate can be regarded as substantial physiological indicators, given their correlation with the physiological demands of pilots. These indicators can serve as a foundation for evaluating cabin temperature control systems.

The experimental results show that the effects of different cabin temperature environments on human physiological response and cognitive function are in line with the expectations in Table 1, which proves that the core temperature limit given by the Mechanical System Coordination Working Group is also applicable to typical people in China. Therefore, physiological indexes with significant differences can be used as safety indexes to evaluate airworthiness compliance. The results of this experiment prove that we can consider simulating the cabin environment temperature, predicting the changes of human core temperature, and measuring the changes in significant physiological indexes to determine the safety and suitability of the cabin environment, and form a method to verify the airworthiness compliance of the cabin high-temperature environment.

### 4.2. Advances and Applications of Physiological Sensors

A variety of physiological monitoring tools were utilized in this study to assess the physiological responses of participants under different heat load conditions. In recent years, significant advancements have been made in physiological monitoring technologies, particularly in the domain of wearable devices and sensor technologies. Garcia proposed a non-invasive method to measure heart rate and skin temperature using mobile biosensors in real-world environments and simulate the computation of the human core temperature in a single pass [18]. Nile designed an Internet of Things (IoT)-based heart rate monitoring system for enhanced flight safety by means of a highly sensitive heartbeat sensor for data input and real-time monitoring at the other end [19]. Future research endeavors can build on this foundation and utilize larger samples and more advanced physiological monitoring techniques to further explore the effects of heat load on different populations. This will provide a more solid theoretical foundation and practical guidance for research in related fields.

### 4.3. Limitations

This study is not without its limitations. First, although the experimental conditions were developed in accordance with FAR 23.831(g), the primary subject group targeted was pilots. Given the specificity of the pilot population, which is predominantly male, an all-male sample was selected. This decision may have precluded the exploration of female responses under similar conditions, thereby limiting the generalizability of the findings and their applicability to different gender groups.

Secondly, the limited sample size, with only 10 individuals in each group, totalling 30, may have compromised the statistical validity and reliability of the results. The limited sample size may have led to instability in the results, particularly in the analysis of gender differences and other underlying variables. Consequently, it is imperative for subsequent studies to encompass a more extensive sample size, inclusive of female subjects, in order to achieve a more profound comprehension of the impact of the heat environment on pilots of diverse genders. This approach will enhance the reliability of the findings and provide more instructive conclusions for practice in related fields.

Finally, in this study, we were unable to conduct our experiments in a high-temperature simulation room combined with a flight simulator due to technical limitations. This constraint may have impacted our ability to collect comprehensive data on flight parameters and emergency decision-making. To address this shortcoming, we opted to evaluate several metrics, including memory, attention, and perceptual ability, as alternatives. However, these metrics may not fully capture the emergency decision-making capabilities of pilots in a real flight environment. Therefore, future research should focus on developing more advanced experimental facilities that integrate high-temperature simulation with flight simulation to more comprehensively assess the effects of high temperatures on pilots.

## 5. Conclusions

According to the results of this experiment, with the increase in environmental temperature, the core body temperature of the subjects gradually increased, reaching 38 °C after about 40 min. At the end of the experiment, the core body temperature remained at about 38.5 °C, and the skin temperature also increased to 36.5 °C. In addition, it was observed that the heart rate of all the subjects increased, and the arterial oxygen saturation and venous oxygen saturation decreased. These results show that the high-temperature environment has some adverse effects on the occupants, but under the experimental conditions, these effects are still within the range of human physiological tolerance. Passengers and crews can try to reduce the adverse effects of high temperature by adjusting their behavior [20]. Under different core temperature–time conditions, the neurobehavioral ability indexes of each age group did not show significant changes. Therefore, the influence of the core temperature limit given by the mechanical system coordination working group on the physiological load and behavioral ability of pilots is within a safe range, and it is also applicable to pilots in China. This experiment shows that the method of external environment temperature–human core temperature–physiological index–safety index can be used as the equivalent safety criterion of CCAR25.831(g) to prove the airworthiness compliance of a cockpit in a high-temperature environment. This study can provide a theoretical basis for the airworthiness verification of civil aircraft in a high-temperature environment.

## Figures and Tables

**Table 1:** Influence of cockpit high-temperature environment on personnel.

38.0~38.5 °C (The Duration Does not Exceed 20 min)		
Personnel	Physiological reaction	Cognitive function
crew	safe	Feel unwell
passenger	safe	Feel unwell

**Table 2:** Changes in Tcore under different levels of heat load.

GROUP	20–30th	30–40th	Over 40th
Before the experiment	37.20 ± 0.16	37.08 ± 0.13	37.23 ± 0.18
38 °C	38.00 ± 0.00	38.00 ± 0.00	38.00 ± 0.00
38~38.5 °C 20 min	38.43 ± 0.10	38.37 ± 0.17	38.43 ± 0.10
F	331.75	300.79	235.06
*p*	0.000 **	0.000 **	0.000 **

** Indicates *p* < 0.01, significance level α = 0.05.

**Table 3:** TSK relational equation summary.

	Sum of Squares	F	Significance *p*	R	R^2^
Regression	150.4540379	246.4837873	0.000	0.847	0.718

**Table 4:** HR relational equation summary.

	Sum of Squares	F	Significance *p*	R	R^2^
Regression	35711.166	174.794	0.000	0.802	0.643

**Table 5:** Fatigue degree relational equation summary.

	Sum of Squares	F	Significance *p*	R	R^2^
Regression	1134.760	250.584	0.000	0.849	0.721

## Data Availability

Due to privacy restrictions, the data used in this study cannot be provided for submission. We assure that the research methodology and findings presented in this paper adhere to ethical standards and have been conducted in accordance with established research protocols.

## References

[1] Wang X. (2022). Ergonomic Design and Performance Evaluation of Individual Cooling Suits for Pilots. Master’s Thesis.

[2] Yao H. (2023). Study on the Influence of Thermal and Acoustic Environment in Deep Underground Space on Workers’ Physiological and Psychological Stress. Master’s Thesis.

[3] Zhang C., Tang S., Li D. (2015). Experimental study on labor load and fatigue level of personnel in high temperature and high humidity environment. J. Saf. Environ..

[4] (2011). Airworthiness Standard for Transport Aircraft Civil Aviation Administration of China.

[5] Zhou B., Chen B., Liu J., Ao Y., Ding L. (2023). Effect of ambient temperature and humidity on muscle fatigue of pilots. Soc. Occup. Ergon..

[6] ARAC (2003). Mechanical Systems Harmonization Working Group (MSHWG) Final Report on FAR/JAR 25.831(g).

[7] Sheng Y. (2008). Study on the Influence of High Temperature Thermal Radiation Environment on Human Physiological Indexes and Tolerance. Master’s Thesis.

[8] Zhao J. (2022). Study on Prediction and Analysis of Human Thermal Regulation Model in Aircraft High and Low Temperature Environment. Master’s Thesis.

[9] Thomas M.M., Cheung S.S., Elder G.C., Sleivert G.G. (2006). Voluntary muscle activation is impaired by core temperature rather than local muscle temperature. J. Appl. Physiol..

[10] Cramer M.N., Gagnon D., Laitano O., Crandall C.G. (2022). Human temperature regulation under heat stress in health, disease, and injury. Physiol. Rev..

[11] Chiles W.D., Iampietro P.F., Higgins E.A. (1972). Combined effects of altitude and high temperature on complex performance. Hum Factors.

[12] Zhou B., Ding L., Chen B., Shi H., Ao Y., Xu R., Li Y. (2021). Physiological Characteristics and Operational Performance of Pilots in the High Temperature and Humidity Fighter Cockpit Environments. Sensors.

[13] Niu J., Hong B., Geng Y., Mi J., He J. (2020). Summertime physiological and thermal responses among activity levels in campus outdoor spaces in a humid subtropical city. Sci. Total Environ..

[14] Zhang H. (2023). Failure analysis of high cockpit temperature in Boeing 737NG aircraft. Aviat. Maint. Eng..

[15] Nag P.K., Ashtekar S.P., Nag A., Kothari D., Bandyopadhyay P., Desai H. (1997). Human heat tolerance in simulated environment. Indian J. Med. Res..

[16] Liu C., Li G., He Y. (2020). Influence of thermal discomfort on human physiological response and work efficiency. Build. Therm. Energy Vent. Air Cond..

[17] Xu B. (2017). Study on the Influence of Cockpit Air Microenvironment on Pilot’s Work Efficiency. Master’s Thesis.

[18] Garcia E., Ferguson D., Napoli N. (2022). Estimating Core Body Temperature Under Extreme Environments Using Kalman Filtering. AIAA SCITECH 2022 Forum.

[19] Muralikumar N., Ramanan N., Rajasekaran M., Kalimuthukumar S. (2023). IoT-Based Heart Rate Monitoring System Designed For Pilots. Proceedings of the 2023 International Conference on Energy, Materials and Communication Engineering (ICEMCE).

[20] Zhang Y., Li J., Liu J. (2020). Experimental study of the impact of passenger behavior on the aircraft cabin environment. Sci. Technol. Built Environ..

